# CFD simulation of CO_2_ absorption by water-based TiO_2_ nanoparticles in a high pressure stirred vessel

**DOI:** 10.1038/s41598-021-81406-1

**Published:** 2021-01-21

**Authors:** Nayef Ghasem

**Affiliations:** Department of Chemical and Petroleum Engineering, United Arab Emirates University, P.O. Box 15551, Al Ain, UAE

**Keywords:** Chemical engineering, Climate sciences, Environmental sciences, Chemistry, Energy science and technology, Engineering

## Abstract

This work presents the modeling and simulation of CO_2_ capture by a water-based Titanium dioxide (TiO_2_) solid nanoparticle in a stirred high-pressure vessel at a constant temperature. Photocatalytic material such as TiO_2_ has excellent properties, namely it is nontoxic, inexpensive, and non-polluting. CFD model equations are developed and solved using COMSOL software package. The effect of the concentration of a solid nanoparticle in a water-based TiO_2_ solution, the size of TiO_2_ nanoparticles and the rate of mixing on the CO_2_ absorption rate is investigated. A 2D mathematical model considers both shuttle and micro-convention mechanisms. Results reveal that the best TiO_2_ concentration range is between 0.5 and 1 kg/m^3^ and that a particle size of 10 nm is more efficient than higher particle sizes. A moderate mixing rate maximizes the CO_2_ removal rate. The theoretical predictions are validated using lab experimental data and those in the available literature. Results confirm that the model calculations match with the experimental results. Accordingly, the model successfully predicts the experimental data and can be used for further studies.

## Introduction

Mass transfer mechanisms of chemical and physical absorbents control the absorption performance of CO_2_ from a flue gas or natural gas streams^1^. The flue gas diffuses in a chemical solvent (such as monoethanolamine), followed by chemical bonding. By contrast, the flue gas diffuses in a physical solvent (such as pure water) and the CO_2_ dissolves in the solvent. Adding nanomaterial to the base fluids (chemical or physical) affects only the diffusion rate^2^. The system operating conditions highly influence the selection of the type of base solvent. Physical solvents are appropriate at high pressure and low temperature while low pressure, a low CO_2_ concentration and a moderate temperature are suitable for chemical-based fluids^3^.

Chemical solvents such as monoethanolamine (MEA), diethanolamine (DEA), and triethanolamine (TEA) are traditional solvents used in the capture of CO_2_ from natural gas and flue gases. However, the integration of amines in CO_2_ capturing processes is subject to several disadvantages, such as: solvent loss due to high volatility; equipment damage as a result of their corrosive nature; and high-energy requirements for the regeneration process. As an alternative solvent, there is focus on a combination of traditional solvents with other components such as ionic liquids^4^, and solid nanoparticles dispersed in a traditional solvent-based fluid to obtain absorbents with enhanced absorbent properties^5^. Methods of removing CO_2_ from a gas stream include absorption, adsorption, as well as membrane and cryogenic methods. Absorption processes are widely used^6^. At normal pressure, the rate of chemical absorption is higher than physical absorption. However, there are several drawbacks; for instance, a large amount of energy is consumed during the regeneration process due to the strong chemical bonds between absorbents and CO_2_^7^. By contrast, the energy consumed during the regeneration of physical absorbents is lower than that used for the regeneration of chemical absorbents^8^. Physical absorption has a low CO_2_ absorption performance compared to chemical absorbents^9^. Consequently, amine-based solvents are prepared as 20–40 wt% aqueous solutions. Considering these facts, the type of solvent used is one of the most important factors in determining the effectiveness and overall dynamics of the carbon dioxide removal process. Much interest is being drawn towards developing new types of solvents that are efficient in the removal process as well as cost effective. Nanofluids are among the alternatives that could possibly achieve results that are competitive with those of industrially-used chemical absorbents such as aqueous alkanolamine. In the absorption of CO_2_ from a gas mixture, there are important factors that affect carbon dioxide capture via nanofluids, including: the type, concentration and size of nanoparticles; and the temperature, pressure and inlet concentration of carbon dioxide^10^. CO_2_ absorption through a microporous membrane process, and comparing two types of nanoparticles, namely silica (SiO_2_,) and carbon nanotubes (CNTs) in water as a base fluid, was studied by Golkhar et al.^11^. The feed consisted of a mixture of air and CO_2_. Results revealed that CNT had a better performance with an absorption efficiency reaching 20% and 40% at high and low liquid flowrates, respectively. A study of the removal efficiency of CO_2_ at two different concentrations of the nanoparticles, namely 0.25 wt% and 0.5 wt%, found that, for both cases of SiO_2_ and CNT nanofluids, the removal efficiency was positively affected by the increase in concentration of nanoparticles^12^. Darabi et al.^13^ obtained similar results for a comparison between CNT and SiO_2_ nanofluids with enhancement values of 32% and 16%, respectively. Results were obtained via modeling and simulation in a membrane module. Rezakazemi et al.^14^ evaluated the absorption effectiveness of nanofluids in a membrane contactor using a 2D mathematical model. The nanoparticles of interest in this study were CNT and SiO_2_ in water. For a concentration range of 0.06–24 wt%, a decrease in the percentage of CO_2_ separation was observed for both CNT and SiO_2_. Jiang et al.^15^ studied four nanoparticles, namely: silica (SiO_2_,), titanium oxide (TiO_2_,) magnesium oxide (MgO) and aluminum oxide (Al_2_O_3_), as well as two base fluids, namely monoethanolamine (MEA) and diethanolamine (MDEA), through a bubbling reactor. Results revealed that absorption of CO_2_ by the nanoparticles was better in MDEA compared to MEA. The experimental enhancement factor at specific nanoparticle loading of 0.1 wt % was found to be 0.99, 1.07, 1.09, and 1.29 using SiO_2_, Al_2_O_3_, MgO, and TiO_2_, respectively. The work by Jiang et al.^15^ also noticed an optimum solid loading in terms of the effectiveness of the process; in particular, the enhancement factor increased to a maximum value, then subsequently decreased for TiO_2_ and Al_2_O_3_. The range of studied concentrations was 0.2–1 kg/m^3^. Peyravi et al.^16^ examined nanoparticles of Fe_3_O_4_, CNT, SiO_2_ and Al_2_O_3_ in water through a pilot-scale membrane contactor. The percent enhancements of the CO_2_ absorption utilizing various nanofluids, namely Fe_3_O_4_, CNT, SiO_2_, and Al_2_O_3_, were 43.8%, 38.0%, 25.9%, 3.0%, with optimum nanoparticle concentrations 0.15 wt%, 0.1 wt%, and 0.05 wt%, and 0.05 wt%, respectively. The different nanoparticles showed different absorption behaviors. Fe_3_O_4_ had the best result while Al_2_O_3_ had a maximum efficiency of only 3% at 0.05 wt% concentration. Rahmatmand et al.^17^ tested the same nanoparticles (Fe_3_O_4_, CNT, SiO_2_, and Al_2_O_3_) in the same base fluid as Peyravi et al.^16^. CO_2_ absorption by Al_2_O_3_ nanoparticle in an NaCl aqueous solution was investigated^18^.

Haghtalab et al.^19^ performed the experiment in a stirred high pressure cell at a constant temperature and concluded that the ZnO nanofluid is more efficient than SiO_2_ nanofluid with water as a base fluid. They studied the effect of the ZnO concentration on the absorption of CO_2_ at 0.05 wt%, 0.1 wt%, 0.5 wt% and 1 wt% at different pressures (1–22 bar) and noticed that, at the same pressure, the effectiveness of the CO_2_ absorption decreases with the concentration. The reasoning behind such a result is attributed to the aggregation of the particles caused by the increase in concentration of the nanoparticles whereby, as a result, less CO_2_ is absorbed. Zhang et al.^20^ evaluated TiO_2_ nanoparticles in a stirred cell and found an optimum value for the concentration of TiO_2_ in a propylene carbonate-based fluid at which the enhancement factor was the highest, by covering the range of 0.6–1.4 kg/m^3^. In the study conducted by Irani et al.^21^, the nanoparticle graphene-oxide (GO) was synthesized and used in MDEA in a process of gas sweetening. It was shown that this absorbent mixture has favorable CO_2_ absorption behavior since GO is characterized by its high surface area and the presence of hydroxide (OH) groups on the surface of the particles. Little change was observed in the absorption enhancement by increasing the concentration of GO from 0.1 to 0.2 wt%. Through a numerical approach while neglecting agglomeration, Koronaki et al.^22^ concluded that the effectiveness of CO_2_ removal increases with the increase in a CNT’s equivalent diameter. In contrast, Zhang et al.^20^ found that, when the concentration of TiO_2_ is low, the effectiveness of CO_2_ removal progressively decreases with an increase in the diameter of the particles while, at higher concentrations, an increase in the size of particles leads to a gradual increase in the enhancement factor. The suggested mechanism behind such an observation is that when the concentration of the particles is low, smaller particles means that more particles are present in the nanofluid that takes up the gas. In contrast, when the concentration is higher, larger particles suggests the presence of a smaller number of particles within the solution and, as a result, viscosity is decreased, which gives a better CO_2_ capture process. Darvanjooghi et al.^23^ used a bubble column to evaluate the effects of the nanoparticle size on the capture of CO_2_. In this study, a mixture of silica-water is used. The tested particle sizes are: 10.6, 20, 38.6 and 62 nm. At the same concentration of silica particles (0.01 wt%), the increase in particle size increased the rate of CO_2_ removal as well as the mass transfer coefficients.

Farzani Tolesorkhi et al.^24^ investigated the removal of CO_2_ by silica nanofluid in water in a cell with no stirrer. It was observed that although increasing the temperature (from 35 to 45 °C) increases the carbon dioxide’s diffusion coefficient in water, the adsorption rate decreases. Pineda et al.^25^ studied the removal rate of CO_2_ by nanofluids in an annular contactor at low rotational speeds. Three nanoparticles in a methanol-based solvent, namely Al_2_O_3_, SiO_2_ and TiO_2,_ were studied. The nanofluids achieved better absorption enhancements in the counter-current flow configuration. The addition of trays further improved the absorption rate for all nanofluids. Kim et al.^26^ investigated the mass transfer through the removal of CO_2_ via a bubble absorption and diffusion process for Al_2_O_3_ in a methanol-based solvent. The Al_2_O_3_ particles positively affected the absorption rate of CO_2_ while the viscosity increased by 11% at a particle concentration of 0.01 vol%. The influence on the surface tension was insignificant. Jorge et al.^27^ aimed to study amine-functionalized multiwall carbon nanotubes (MWCNTs) in water. While enhancing CO_2_ absorption, the amine functional groups also increases the hydrophilicity of the MWCNTs, which enables the particles to remain suspended in water for long periods (at least three months at room temperature). Compared to pure water, the absorption capacity of these MWCNTs is 36% higher at a particle concentration of approximately 40 mg/L.

The present work studies the possible enhancement of CO_2_ absorption by water-based TiO_2_ solid nanoparticles in a high-pressure stirred cell. The study investigates the influence of TiO_2_ loading, particle size and mixing rate on the CO_2_ absorption rate. A transient 2D mathematical model is developed to describe and predict the CO_2_ pressure drop and absorption rate in the high-pressure stirred cell at a constant temperature.

## Experimental

**Figure 1:**
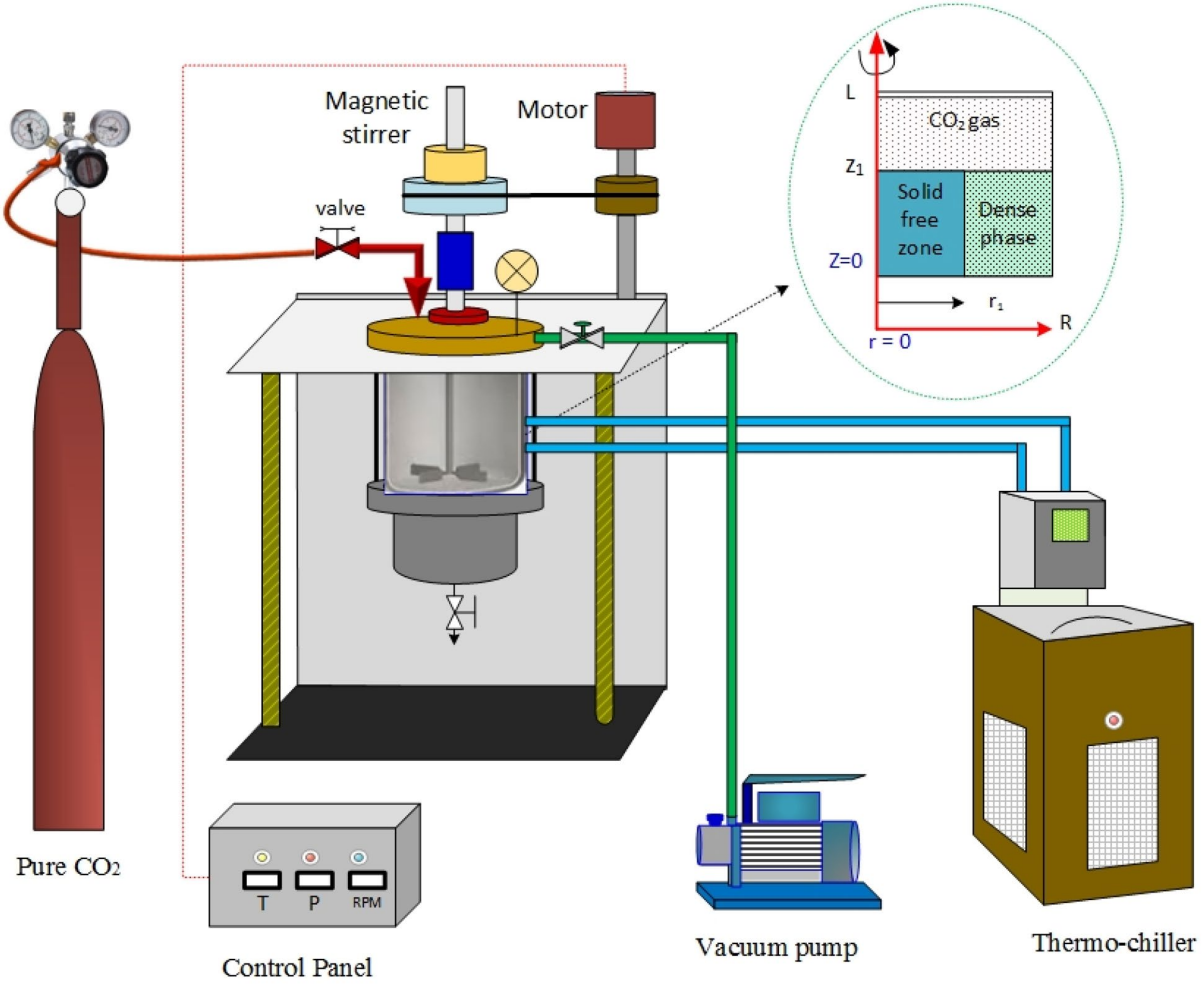
Schematic of the experimental setup and the modeling zone used for the absorption of CO_2_ by nanofluid in a high-pressure stirred cell. Figure generated using Microsoft Visio Professional 2016 (Microsoft.com).

Figure 1 is a schematic diagram of the experimental setup used to measure the CO_2_ pressure in a high-pressure stirred cell. A precise volume of water-based TiO_2_ nanoparticles was added to the cell and a vacuum pump evacuated the cell’s empty space. Pure CO_2_ gas filled the displaced space of the cell. The controller of the stirred cell recorded the temperature and pressure, and manipulated the rotation speed of the magnetic stirrer. The chiller controlled the stirred cell temperature by circulating water in the jacket of the cell. The vacuum pump removed any air or gas above the surface of the liquid as well as any gas bubbles in the nonabsorbent fluid. The nanofluid was prepared by mixing a specific amount of TiO_2_ solid nanoparticles (size 10 nm) dispersed in 500 ml of water. A high intensity ultrasonic liquid processor was used for at least 30 min to form a homogenous solution.

## Model development

A dynamic 3D mathematical model considers both micro-convection and a shuttle mechanism in a cylindrical coordinate system (*r*, *z*, *θ*). The model is employed to depict a concentration profile in radial, axial and angular directions. The micro-convections described by the Brownian movement of the nanoparticles cause fluctuations of the liquid around the nanoparticles. Accordingly, the liquid–gas mass transfer is enhanced due to the convective mass transfer in the bulk of the liquid. By contrast, the shuttle mechanism resulting from the movement of the nanoparticle to and from the liquid–gas interface absorbs the gas and desorbs it to bulk the liquid (regeneration of nanoparticles). Hence, the continuous movement of nanoparticles between the bulk liquid and liquid–gas interface enhances the mass transfer. It is assumed that the nanoparticles are of spherical shape and surrounded by a liquid layer, due to the small size of nanoparticles, *the mass transfer resistance inside the particles is neglected.*. In order to simplify modeling of the process, the system is divided into three sub areas: gas, liquid and dense phase regions. The model equations were solved using finite element methods built in the efficient COMSOL Multiphysics software package^28^.

### Gas phase region

The transport of CO_2_ from the gas phase to the nearby liquid and dense phases is by diffusion and is described by Eq. (1):

1 $$\frac{{\partial C_{Ag} }}{\partial t} = D\left( {\frac{1}{r}\frac{\partial }{\partial r}\left( {r\frac{{\partial C_{Ag} }}{\partial r}} \right) + \frac{1}{{r^{2} }}\frac{{\partial^{2} C_{Ag} }}{{\partial \theta^{2} }} + \frac{{\partial^{2} C_{Ag} }}{{\partial z^{2} }}} \right)$$

The arbitrary boundary conditions are:

2 $$\begin{aligned} & {\text{At}}\quad z = z_{1} \quad N_{A} = - k_{l} \left( {C_{Ag} - C_{AL} } \right)\quad \left( {{\text{molar}}\,{\text{flux}}} \right) \\ & {\text{At}}\quad z = L \frac{{dC_{Ag} }}{dz} = 0\quad \left( {{\text{convective}}\,{\text{flux}}} \right) \\ & {\text{At}}\quad r = R \frac{{dC_{Ag} }}{dr} = 0\quad \left( {{\text{convective}}\,{\text{flux}}} \right) \\ & {\text{At}}\quad r = 0 \frac{{dC_{Ag} }}{dr} = 0\quad \left( {{\text{axial}}\,{\text{symmetry}}} \right) \\ \end{aligned}$$

The initial conditions:

at $t = 0\, C_{Ag} = C_{Ag0}$ (initial concentration of CO_2_ in the cell above the liquid solvent nanofluid).

### Liquid phase region

The mass transport of CO_2_ in the liquid phase is defined by the component balance equation in the cylindrical coordinate:

3 $$\frac{{\partial C_{AL} }}{\partial t} = D\left( {\frac{1}{r}\frac{\partial }{\partial r}\left( {r\frac{{\partial C_{AL} }}{\partial r}} \right) + \frac{1}{{r^{2} }}\frac{{\partial^{2} C_{AL} }}{{\partial \theta^{2} }} + \frac{{\partial^{2} C_{AL} }}{{\partial z^{2} }}} \right)$$

where the concentration of CO_2_ in the liquid phase is *C*_*AL*_,

4 $$\begin{aligned} & {\text{at}}\quad z = z_{1} \quad N_{A} = k_{l} \left( {C_{Ag} - C_{AL} } \right)\quad \left( {{\text{molar}}\,{\text{flux}}} \right) \\ & {\text{at}}\quad z = 0\quad \frac{{dC_{AL} }}{dz} = 0\quad \left( {{\text{convective}}\,{\text{flux}}} \right) \\ & {\text{at}}\quad r = r_{1} \quad C_{AL} = C_{Ad} \\ & {\text{at}}\quad r = 0\quad \frac{{dC_{AL} }}{dr} = 0\quad ({\text{axial}}\,{\text{symmetry}}) \\ \end{aligned}$$

### Dense phase region

The CO_2_ mass transfer in the dense phase is described by the following equation:

5 $$\frac{{\partial C_{Ad} }}{\partial t} = D\left( {\frac{1}{r}\frac{\partial }{\partial r}\left( {r\frac{{\partial C_{Ad} }}{\partial r}} \right) + \frac{1}{{r^{2} }}\frac{{\partial^{2} C_{Ad} }}{{\partial \theta^{2} }} + \frac{{\partial^{2} C_{Ad} }}{{\partial z^{2} }}} \right) - h\frac{{A_{s} }}{{V_{s} }}\left( {C_{AL} - C_{As} } \right)$$

The suitable boundary conditions are:

6 $$\begin{aligned} & {\text{At}}\quad z = z_{1} \quad N_{A} = k_{l} \left( {C_{Ag} - C_{Ad} } \right)\quad \left( {{\text{molar}}\,{\text{flux}}} \right) \\ & {\text{at}}\quad z = 0\quad \frac{{dC_{Ad} }}{dz} = 0\quad \left( {{\text{convective}}\,{\text{flux}}} \right) \\ & {\text{at}}\quad r = r_{1} \quad C_{Ad} = C_{AL} \\ & {\text{at}}\quad r = R\quad \frac{{dC_{Ad} }}{dr} = 0\quad \left( {{\text{convective}}\,{\text{flux}}} \right) \\ \end{aligned}$$

where *k*_1_ is the mass transfer coefficient in the presence of nanoparticles (m/s) obtained from the experimental data as a function of nanoparticle loading in the base fluid. The concentration of CO_2_ in the liquid dense phase is *C*_*Ad*_, the mass transfer coefficient of the convective phase is *h*, the surface area of one nanoparticle to its volume is *A*_*s*_/*V*_*s*_, and *C*_*AS*_ is the concentration of CO_2_ at the solid surface. The mass transfer of CO_2_ in the dense phase between the liquid and solid particles is achieved by the following equation:

7 $$\frac{\partial q}{{\partial t}} = h\frac{{A_{s} }}{{m_{s} }}\left( {C_{Ad} - C_{As} } \right)$$

where q is the amount adsorbed of CO_2_ per unit mass of solid particles. The adsorption mechanism is described by a Langmuir adsorption isotherm:

8 $$q = q_{m} \frac{{k_{q} C_{As} }}{{1 + k_{q} C_{As} }}$$

Rearranging the equation for *C*_*AS*_

9 $$C_{As} = \frac{{q/\left( {q_{m} k_{q} } \right)}}{{1 - q/q_{m} }}$$

where *q*_*m*_ is the maximum amount of CO_2_ being adsorbed at the surface of the solid nanoparticles.

The trend understood by the nano motion and the Einstein–Stokes equation quantifies the Brownian diffusion of a single particle:

10 $$D = \frac{{k_{B} \cdot T}}{{3\pi \cdot \mu \cdot d_{p} }}$$

where D is the diffusion coefficient, *k*_*B*_ is the Boltzmann constant (1.38 × 10^−23^ m^2^ kg/s^2^K), *μ* is the solvent viscosity, and *d*_*p*_ is the droplet diameter.

The volumetric mass transfer coefficient obtained experimentally for nanofluids is as follows^29^:

11 $$\frac{{V_{G} }}{RT}\frac{{dP_{A} }}{dt} = k_{L} A \left( {\frac{{P_{A} }}{H} - C_{Ad} } \right)$$

where *V*_*G*_ (m^3^) is the gas volume, *P*_*A*_ (Pa) the gas pressure, *H* (m^3^ Pa/mol)) is the Henry coefficient, *C*_*Ad*_ (mol/m^3^) is the solute concentration in the dense phase, and *A* (m^2^) is the liquid–gas contact area.

The mixing rate (*φ*) of the liquid phase is described by the following equation:

12 $$\varphi = \omega \pi$$

where *ω* is the mixing rate constant in (rad/s), and *π* is 3.14.

## Results and discussion

### Effect of nanoparticle concentration

The increase in the concentration of nanoparticles in the base fluid does not essentially improve mass transfer. In certain cases, the increase in nanoparticle concentration decreases the mass transfer at a lower level than the base fluid without nanoparticles^12, 30–32^.

**Figure 2:**
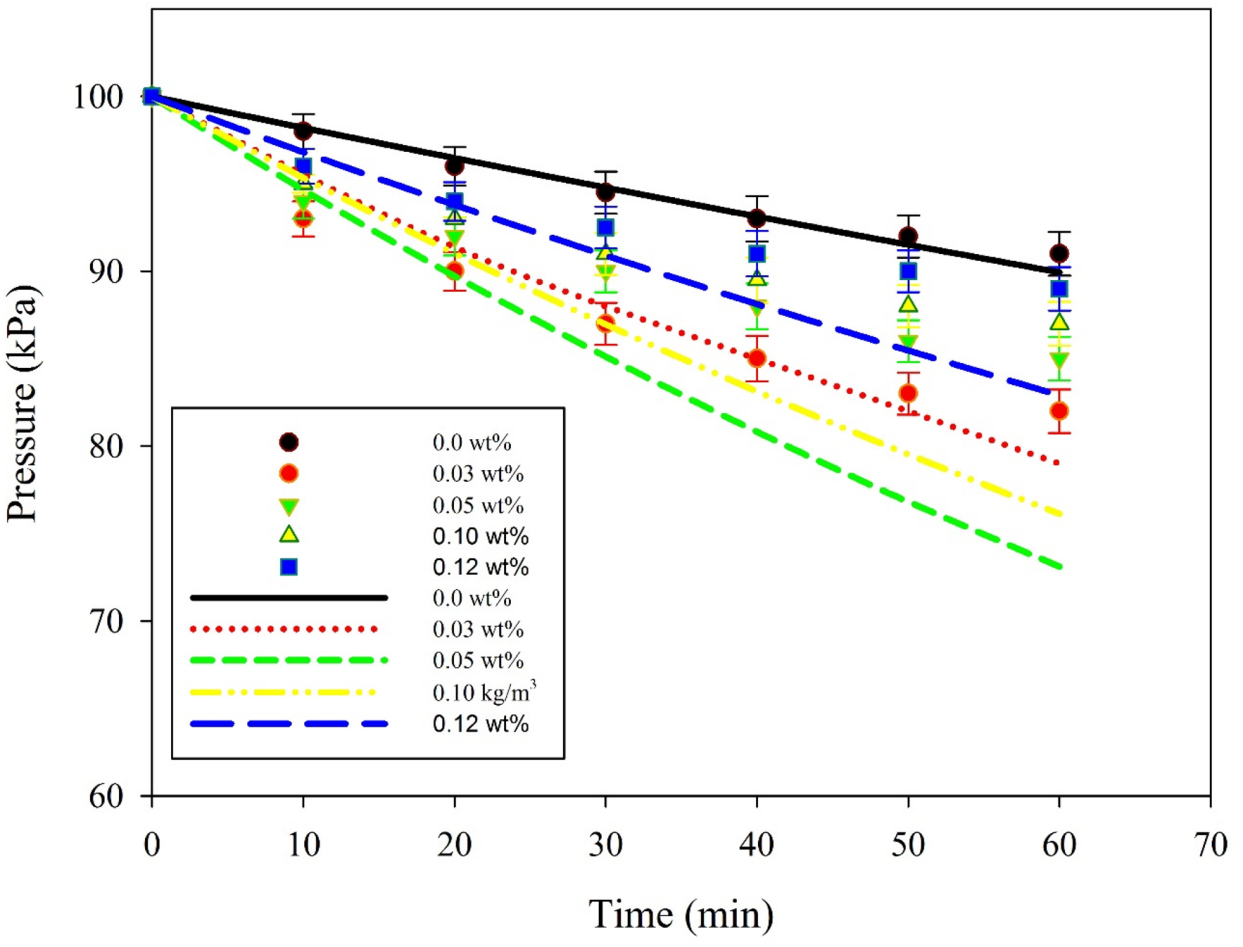
Comparison of experimental data and model predictions for the pressure of CO_2_ versus time for different TiO_2_ loading, particle size 10 nm nanoparticle and 25 °C temperature. The solid lines represent the model predictions. Mixing rate 120 RPM.

Figure 2 illustrates the pressure of carbon dioxide removed with time for variable solid concentrations in base fluids. The size of the nanoparticles used in the study was 10 nm nanoparticles TiO_2_. The results reveal that the solids loadings have strong impact on increasing the rate of the CO_2_ pressure drop. Results also demonstrate that there is a solids loading limit beyond which the CO_2_ absorption rate decreases. This is attributed to the interaction of the dispersed phases. No interaction of nanoparticles occurs at a very low concentration of solid nanoparticles (< 0.005 wt%). Accordingly, the solid nanoparticles move freely. As the solid nanoparticle concentration increases, the effect of convectional mass transfer increases, hence promotes the performance of the CO_2_ absorption rate (in the range of 0.005–1 wt%). By contrast, as the concentration of the solid nanoparticles exceeds a certain value, the free spacing between the solid nanoparticles decreases, which suppresses their interaction and free movement, hence the CO_2_ absorption rate decreases^33^. At high nanoparticle concentration, the distance between dispersed nanoparticles decreases, hindering the movement of particles, hence decreases the local convection. The high solid concentration also reduces the interfacial area between the absorbent nanoparticles and the CO_2,_ hence reduces the absorption rate.

The performance of CO_2_ absorption decreases at high nanoparticle concentration because of the increase in the viscosity of the absorbent nanofluid. Viscosity increases as nanoparticle concentration increases. This increase in viscosity is negligible at low nanoparticle concentrations. The increase of the nanoparticle concentration beyond a critical value slows the Brownian fluid motion due to the interactions of inter particles^34^.

**Figure 3:**
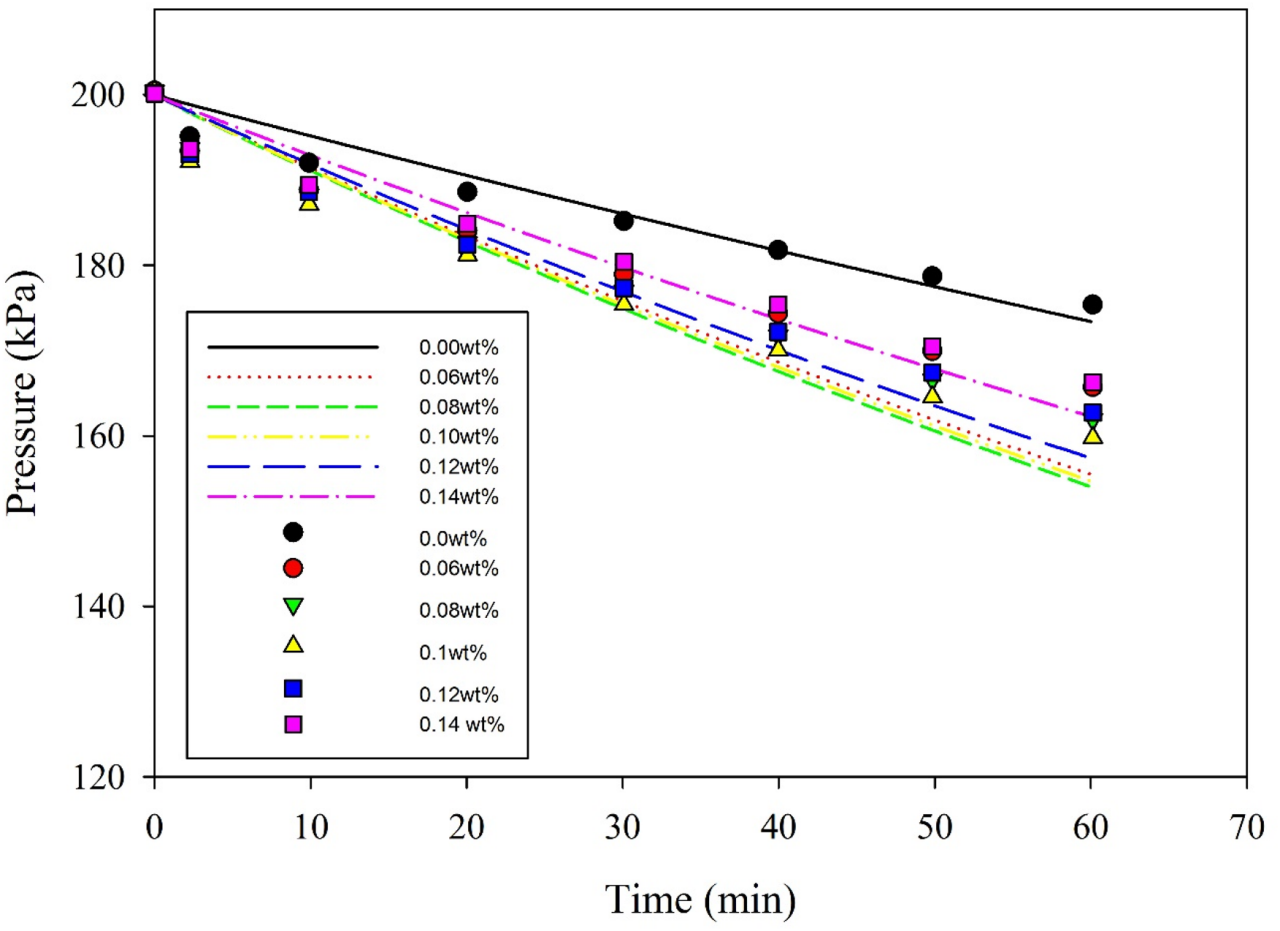
Comparison of developed model predictions (lines) and experimental data (no mixing, 0 RPM) available in the literature^20^ for variable concentrations of TiO_2_ with 10 nm nanoparticles at 25 °C. The solid lines represent the model predictions.

Validation of the developed mathematical model is obtained by comparing the present model predictions with the experimental data obtained from the absorption of pure CO_2_ gas in a high-pressure stirred cell reactor. Figure 3 demonstrates the absorption of CO_2_ without mixing with a propylene carbonate-based TiO_2_ nanoparticle inside the stirred cell^20^. The results were in good agreement with model predictions. Furthermore, the model can be used for investigating the effect of other operating parameters on the CO_2_ removal rate.

**Figure 4:**
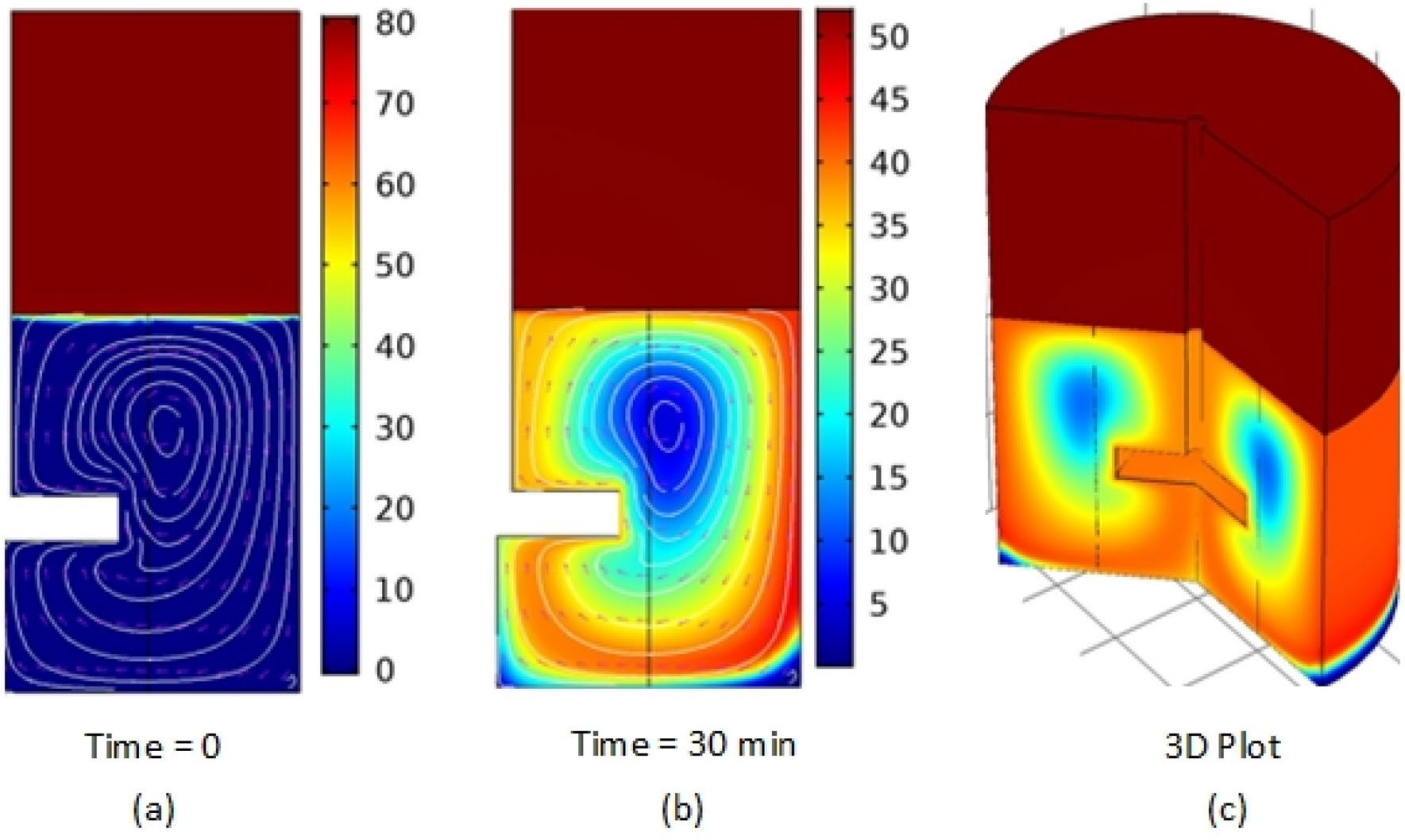
CO_2_ concentration (mol/m^3^) profile in pure water versus time in a high pressure stirred cell. The pressure is atmospheric; temperature is 24 °C; initial CO_2_ concentration is 80 mol/m^3^; the particle diameter is 10 nm, with 0.1 wt particle loading. Image generated using Comsol Multiphysics version 5.5 (comsol.com).

Figure 4 demonstrates the CO_2_ concentration profile throughout the high-pressure stirred cell. The diagram reveals that the CO_2_ concentration in the gas phase is initially 80 mol/m^3.^ With time, the concentration declines to around 50 mol/m^3^ while the concentration of CO_2_ in the liquid phase increases due to the absorption of CO_2_ from the gas phase to the liquid phase. The increased mixing rate homogenizes the concentration profile of the liquid phase. The dead zone at the center of the tank is attributed to the low mixing rate.

### Effect of mixing rate

**Figure 5:**
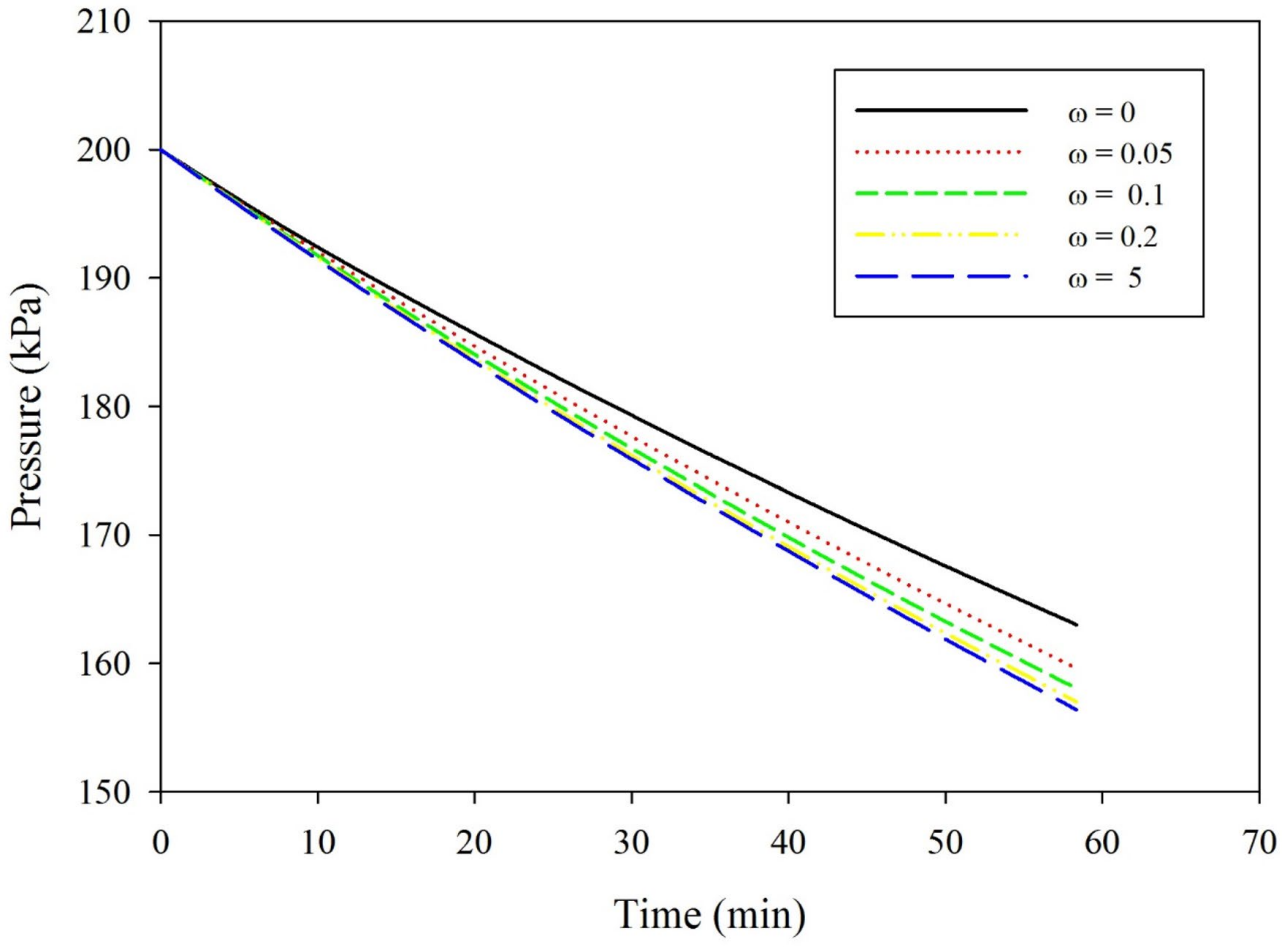
CO_2_ pressure versus time in a stirred cell at various mixing rates; initial pressure 200 kPa; and temperature 25 °C.

Figure 5 shows the effect of mixing rate with time on the CO_2_ pressure drop in the gas phase region of the stirred cell. The gas pressure decreased significantly as the mixing rate increased from *ω* from 0 to 0.1. With further increase in the mixing rate (*ω* = 0.1–0.2), the effect on the pressure removal rate is insignificant. After 60 min of operation, increasing the *ω* value from 0.0 to 0.1 decreases the gas pressure from 171 to 166 kPa. Increasing the *ω* value from 0.1 to 0.2 results in a decrease to 165 kPa. Further increase of *ω* results in an insignificant decrease in the rate of the CO_2_ pressure drop. A high mixing rate results in perfect mixing, hence a homogenous phase where more CO_2_ is being absorbed by the nanofluid. Mixing accelerates the convective motion of the nanoparticles, such as the Brownian motion that forces the nanoparticles to interact with the CO_2_ at the liquid–gas interface, decreases the thickness of the diffusion boundary layer, and assists the CO_2_ gas to diffuse into the bulk base fluid^22^.

### Effect of initial gas pressure

**Figure 6:**
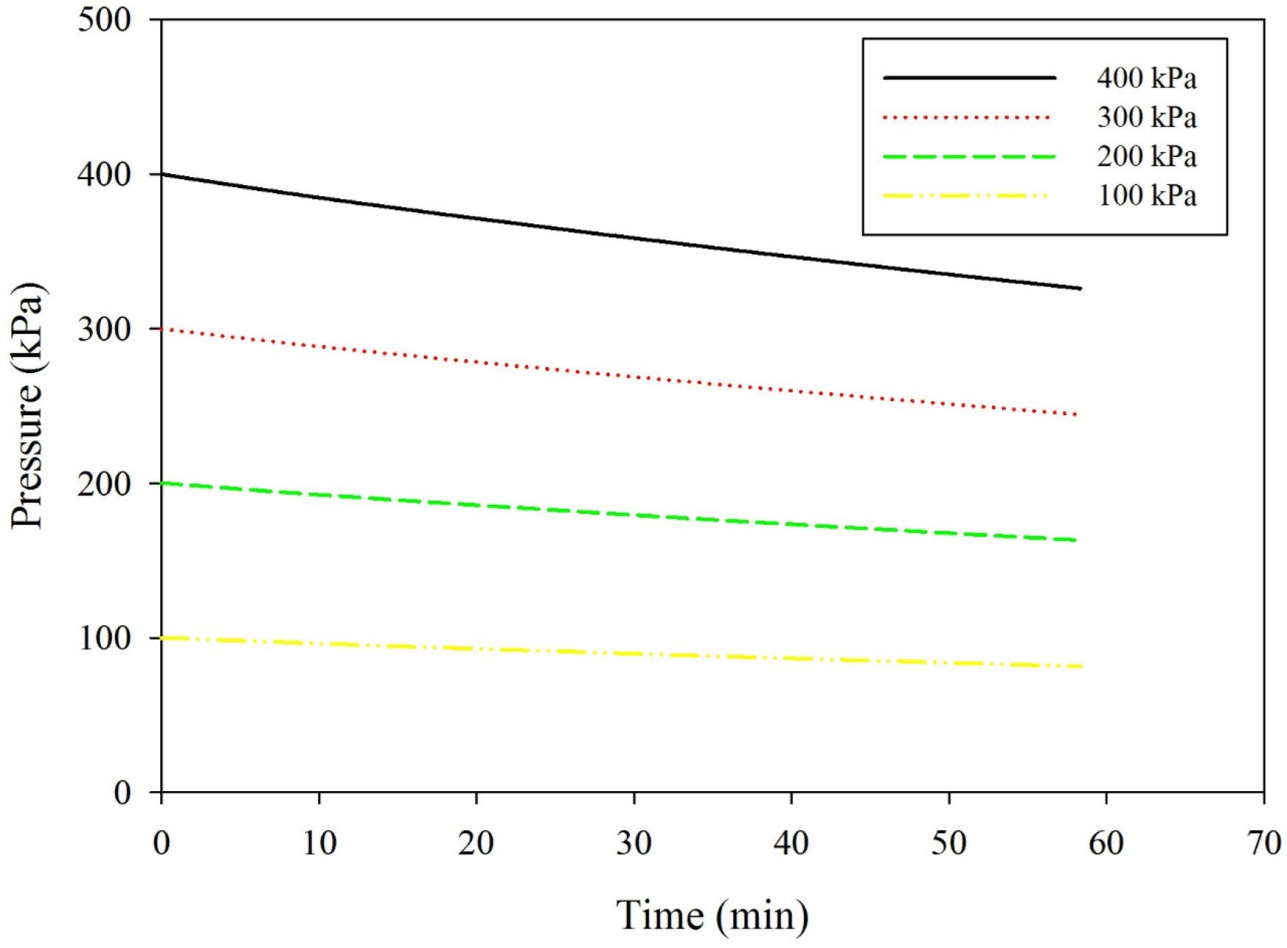
Effect of CO_2_ initial gas pressure versus time on the pressure drop rate under no mixing conditions; temperature is 25 °C, nanoparticle diameter is 10 nm, with 0.1 wt% TiO_2_ solids loading.

**Figure 7:**
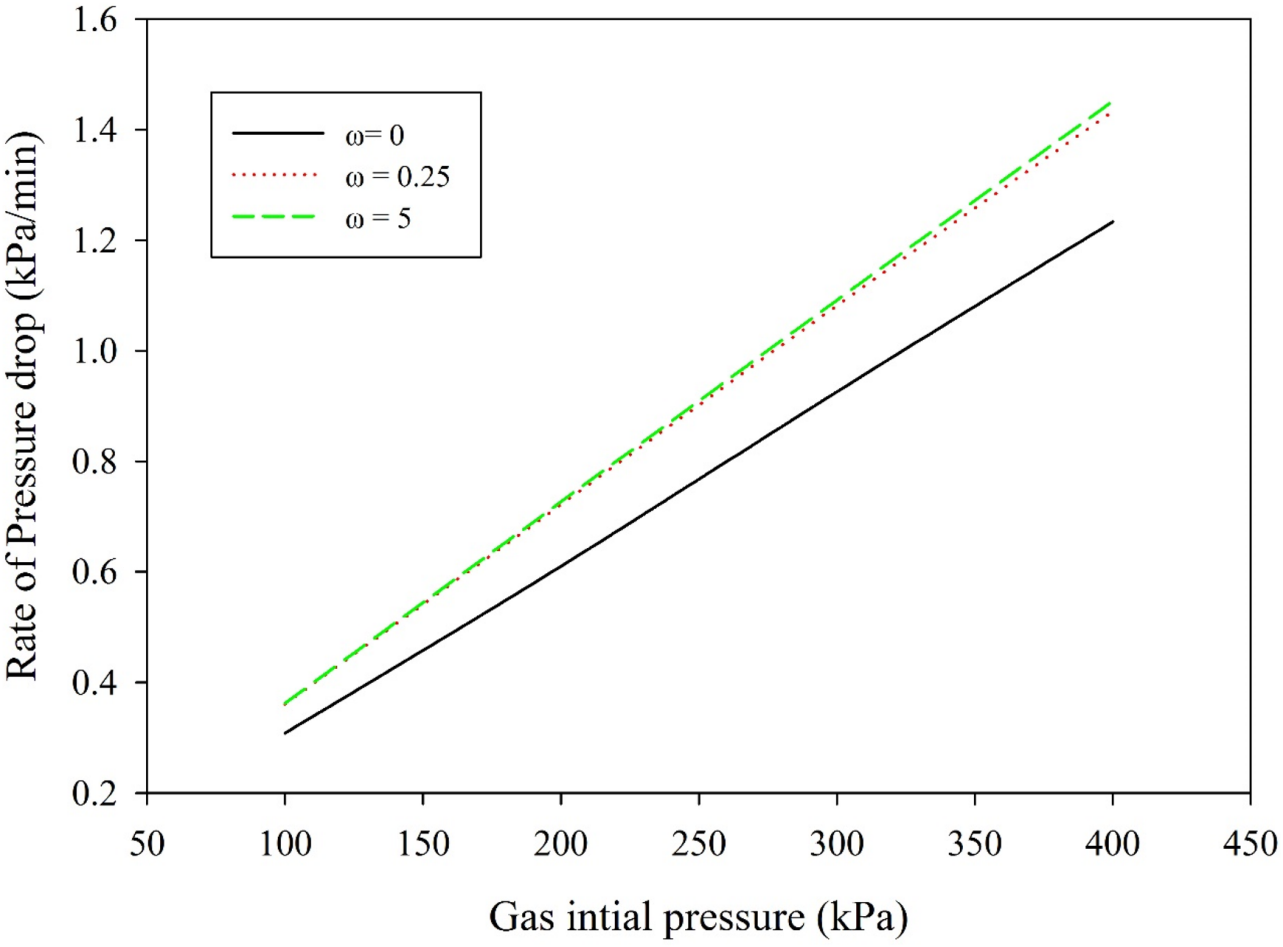
Effect of the initial gas pressure on the rate of CO_2_ pressure drop at variable mixing rates; temperature is 25 °C; nanoparticle diameter is10 nm, with 0.1 wt% TiO_2_ solid loading.

Figure 6 shows the effect with time of the initial gas pressure on the rate of the CO_2_ pressure drop. The results reveal that as the initial CO_2_ gas pressure increases, the rate of the gas pressure drop also increases, as expressed in Fig. 7. The increase in the solubility of the CO_2_ in the nanofluid is attributed to the pressure that increases the concentration gradient, hence increases the CO_2_ removal flux. The rate of decrease in the CO_2_ gas pressure is directly proportional to the initial gas pressure in the gas compartment of the absorption cell. A similar result was observed for the effect of pressure on the CO_2_ absorption performance in the existing nanoparticles^35^. The absorption enhancement of nano absorbent increases with increasing pressure. According to the Einstein-Stokes equation, for most fluids, the viscosity increases monotonically with the pressure, hence hinders the Brownian motion^36^. In contrast, high pressure increases the absorption performance because of the exterior force exerted by the high pressure, which reduces the size of the clusters.

The effect of the mixing rate on the CO_2_ removal rate is illustrated in Fig. 7. The rate of the pressure drop increases with an increased mixing rate to a certain limit. A further increase in the mixing rate has an insignificant effect on the CO_2_ removal rate. According to Henry’s law of solubility, for the physical absorption method, the solubility of the gas increases as the temperature decreases and the pressure increases.

### Effect of nanoparticle size

**Figure 8:**
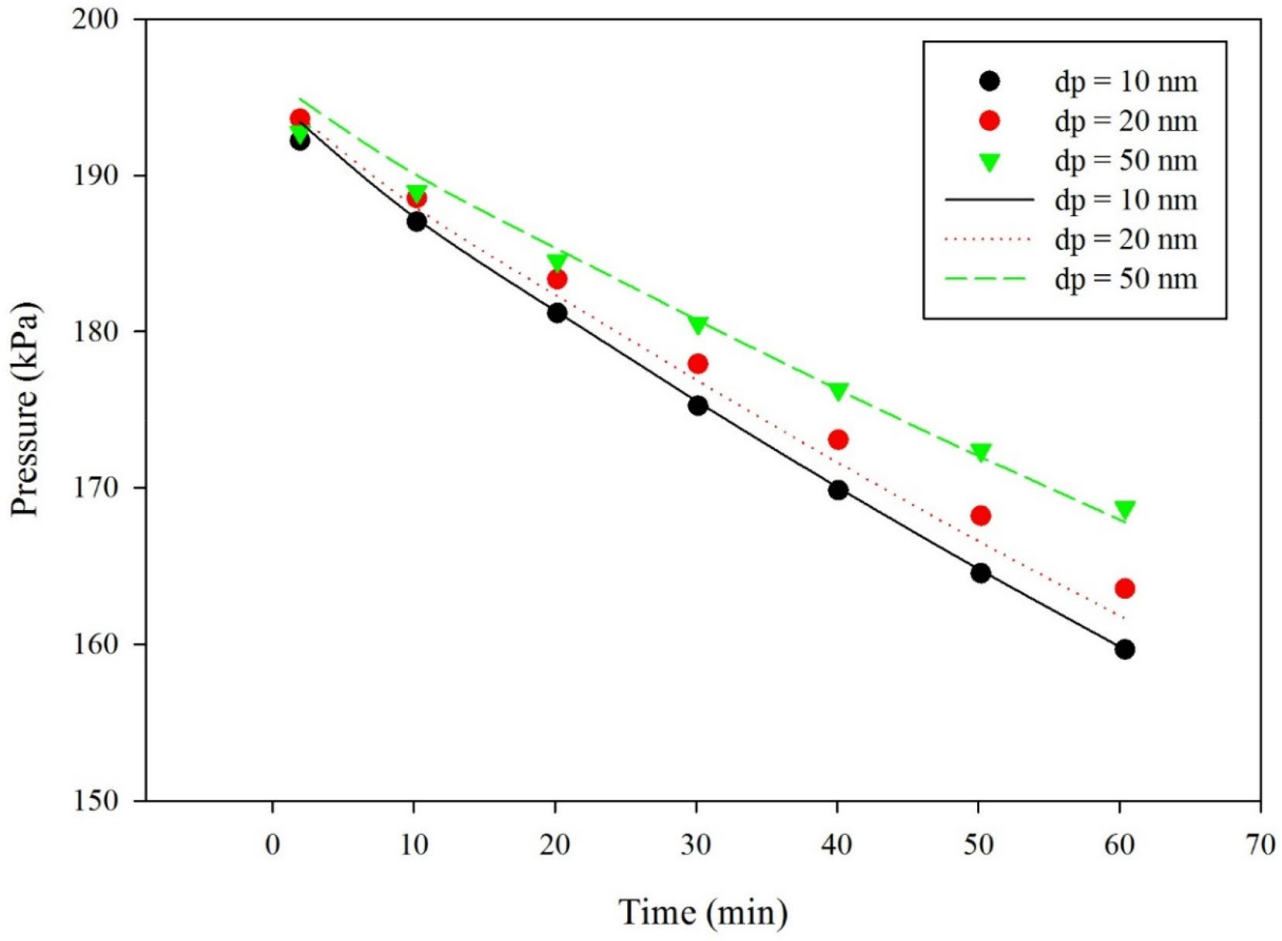
Comparison between model predictions (solid lines) and experimental data^20^ for the change of CO_2_ pressure with time at different TiO_2_ particle size, with 0.1 wt% solid loading and a temperature of 25 °C, 0 RPM.

Figure 8 shows the effect of particle size on the CO_2_ pressure drop at a constant mixing rate of 1 kg/m^3^ TiO_2_ nanoparticle. The figure also illustrates that there is an optimum size of nanoparticle, that is 10 nm, beyond which the absorption rate of CO_2_ declines. According to the Einstein-Stokes equation, the particle size is inversely proportional to the particle diffusion coefficient. The inverse relations between particle size and absorption performance were reported by many researchers^32, 37–39^. Comparison of model predictions and experimental data obtained from the literature^20^ are in good agreement. Particles in nanofluids can move as single or aggregated state. If particles move in aggregated state, their influence on absorption rate is insignificant. This may contribute to the reason for the decrease in absorption performance at high nanoparticle concentration. Similar results were observed by Lee and Kang^18^.

## Conclusion

This paper presents the influence of a water-based TiO_2_ solid nanoparticle on the performance of CO_2_ absorption in a high-pressure stirred cell. The addition of TiO_2_ nanoparticles to classical water-based solvents offers advantages to the overall performance of the base solvents. Nevertheless, the enhancement in the CO_2_ removal process relies on many factors concerning either the added component to the base solvent or the operating mechanism and conditions of the absorption process such as TiO_2_ concentration, mixing rate, size of the nanoparticles. Results reveal that a stirred cell reactor is efficient in CO_2_ removal using TiO_2_ nanoparticles. There is optimum concentration of around 0.1 wt%, beyond which the removal rate declines. There is also an optimum mixing rate and particle size; a low particle size performs more reliably than a large particle size. The developed CFD mathematical agreed well with experimental data at low operating time and no mixing rate. By contrast, discrepancy increased with time and with mixing rate. Moderate mixing rate improves rate of CO_2_ absorption.
